# Infection with an acanthocephalan helminth reduces anxiety-like behaviour in crustacean host

**DOI:** 10.1038/s41598-022-25484-9

**Published:** 2022-12-15

**Authors:** Camille-Sophie Cozzarolo, Marie-Jeanne Perrot-Minnot

**Affiliations:** Biogéosciences, UMR 6282 CNRS, Université Bourgogne Franche-Comté, 6 Boulevard Gabriel, 21000 Dijon, France

**Keywords:** Behavioural ecology, Ecology, Emotion

## Abstract

Trophically transmitted heteroxenous parasites of diverse clades can decrease or reverse antipredator behaviours in their intermediate hosts, thereby increasing their chances of reaching their final hosts. Such behavioural alterations could result from compromised cognitive abilities affecting fear- or more generally stress-related neurophysiological pathways. We tested this hypothesis in a key model system in the study of parasitic manipulation, the fish acanthocephalan parasite *Pomphorhynchus tereticollis* and its intermediate crustacean host *Gammarus fossarum,* using the ‘threat of electric shock’ paradigm. We exposed uninfected and infected *G. fossarum* to chronic and/or acute electric shock programs at two different intensities (voltage), and then quantified their sheltering behaviour as a proxy for anxiety-like state. Infected gammarids did not express anxiety-like response to electric shocks, while uninfected gammarids hid more when exposed to acute treatments, and when exposed to the high intensity chronic treatment. Interestingly, the lack of response in infected gammarids depended on parasite developmental stage. Our results support the hypothesis that this acanthocephalan parasite impacts the general anxiety-like circuitry of their intermediate host. Further studies are needed to investigate whether it involves inappropriate processing of information, impaired integration, or altered activation of downstream pathways initiating behavioural action.

## Introduction

Parasites from a wide diversity of clades cause remarkable changes in their host’s phenotype, including their behaviour (“parasite-induced phenotypic alterations”, PIPA)^1,2^. Some of these phenotypic alterations seem to give parasites a fitness advantage by allowing or facilitating the completion of their life cycle^3–6^, a strategy often termed “manipulation”. Alterations of host behaviour are particularly frequent in heteroxenous, trophically transmitted parasites, with infected hosts showing behaviours demonstrated or supposed to increase their chances to be predated by their parasite’s final host^7–9^. These parasites generally alter the antipredator behaviours of their intermediate hosts, and some parasites are even able to reverse their intermediate host’s fear of predator cues, such as the protozoan *Toxoplasma gondii*^7^ and acanthocephalan parasites^8^. Interestingly, PIPAs do not have to be specific to predators suitable as final hosts to evolve^9^, in particular when predation pressure is low, as shown by^10^. On the other hand, evidence for adaptive specificity according to the parasite’s developmental stage has been provided both experimentally^11^, and theoretically^12^.

Despite their wide occurrence, parasite-induced behavioural alterations are still understudied at the proximate level, and mechanisms of manipulation remain rarely elucidated^13,14^. Threat-evoked behaviours are often the expression of cognitive processes involving non-associative and associative learning. Their flexibility and context-dependent modulation makes them particularly prone to alteration by parasites, possibly through their supporting neuromodulatory systems^15,16^. Therefore, one promising path to unravel the proximate mechanisms of altered defensive behaviour is to investigate whether parasites interfere with their hosts’ cognitive abilities linked with fear and anxiety^17^. Anxiety can be defined as “the suite of anticipatory affective, cognitive and behavioural changes in response to uncertainty about a potential future threat”^18^, while fear responds to existing cues indicative of a threat^19^. Interestingly, some parasite-induced behaviour alterations can be mimicked by anxiolytics, such as the “fatal attraction” to cats of rats infected with *Toxoplasma gondii*^7,20^. In addition, serotonin, a biogenic amine involved in anxiety-like and antipredator behaviours in invertebrates as well as in vertebrates^21,22^, has been identified as a likely target for manipulative parasites in general, and, notably, acanthocephalans^15,23,24^.

Acanthocephalans are trophically transmitted helminths with life cycles generally involving two successive hosts: an arthropod used as intermediate host, and its vertebrate predator, whose digestive tract serves for parasite sexual reproduction. They are studied for their ability to alter their intermediate hosts’—proved or supposed—antipredator behaviours such as changes in micro-habitat preference and cue-triggered defensive or escape responses (reviewed in^25^), in ways that appear to increase parasite transmission. Such alterations seem restricted to the parasite’s developmental stage infective to the definitive host—cystacanth—, while the non-infective younger developmental stage—acanthella—produces either the reverse alteration, or none^11,25^. From a mechanistic point of view, amphipods infected with the fish acanthocephalan parasites *Pomphorhynchus laevis* and *P. tereticollis* have an increased brain serotonin immunoreactivity, which is proportional to the intensity of their phototaxis^26^. Photophobia is thought to decrease predation risk although the direct link between serotonin-induced photophobia reduction and predation rate was not evidenced in uninfected gammarids^27^. Behaviours similar to those of acanthocephalan-infected gammarids (decreased photophobia and geotaxis, but not sheltering behaviour) can be induced by the injection of serotonin in uninfected *Gammarus pulex*^26,28^. *Pomphorhynchus tereticollis* alters its amphipod host in at least two ways: contrary to uninfected individuals, infected *Gammarus fossarum* do not increase their sheltering behaviour in the presence of a bullhead (*Cottus gobio*, predator of amphipods and final host of *P. tereticollis*) and are significantly attracted to bullhead odour^8^. In addition, it is one of the few species of manipulative parasites whose strategy was actually demonstrated to result in higher transmission rates in the wild, as its prevalence was 10 times higher in gammarids found in bullhead stomachs than in living gammarids sampled in the same river^8^.

Here, we aimed to test the hypothesis that *P. tereticollis* alters sheltering behaviour by interfering with the general anxiety circuitry of its intermediate host *G. fossarum*, using the ‘threat of electric shock’ paradigm*.* Gammarids and crayfish ‘tail-flick’ when exposed to electric shocks (ES), which is interpreted as an aversive reaction to noxious stimuli in crustaceans^29–32^. Both associative and non-associative learning has been evidenced in crustaceans using the ES paradigm^33,34^. ES were therefore used as an uncontrollable and unpredictable stressor^35^ that is not ecologically relevant for either host or parasite, in order to evaluate the hypothesis that acanthocephalan parasites interfere with general anxiety circuitry. Sheltering behaviour is considered a proxy for not only fear (e.g. in response to predator cues^8^), but also anxiety in *G. fossarum*, as ES-induced increase of refuge use can be diminished by an anxiolytic treatment (metabotropic glutamate receptor group II/III agonist LY354740^29^). Since acute and chronic stress involve different (neuro-)physiological changes^36^ with which the mechanisms of parasite manipulation might interfere, we administered ES acutely (five minutes before the refuge use test) and/or chronically (daily for six days). The magnitude of behavioural response to stress may also depend on stress intensity. We therefore applied acute and chronic ES at two voltages. We expected uninfected gammarids that were exposed to ES to hide under the refuge more often than non-shocked gammarids, as a response to acute^29^ and chronic stress. This behavioural response should be more pronounced in gammarids exposed to high stress level. Cystacanth(s)-infected gammarids were predicted to react less to stress caused by ES than uninfected gammarids.

## Results

**Figure 1:**
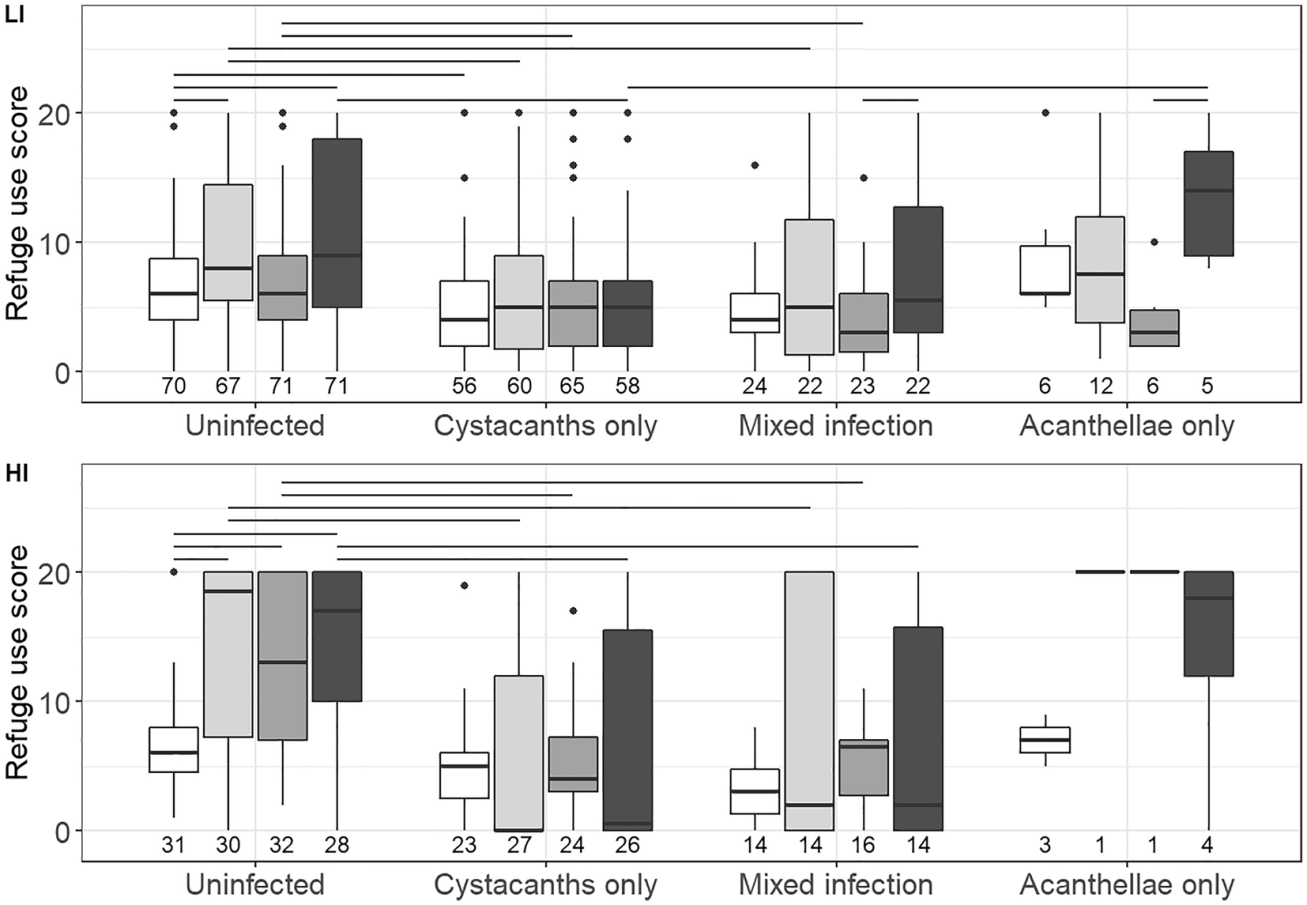
Refuge use of *Gammarus fossarum* according to electric-shock (ES) treatments and infection statuses with the acanthocephalan *Pomphorhynchus tereticollis*, in both experiments. White boxes: controls; light grey boxes: acutely but not chronically stressed; medium grey: chronically but not acutely stressed; dark grey: acutely and chronically stressed gammarids. *LI*: “low intensity” ES treatment; *HI*: “high intensity” ES treatment. Groups differing significantly after Benjamini–Hochberg correction are linked by horizontal bars above boxplots. Only relevant significant comparisons are shown. Numbers below the boxes represent sample sizes.

A total of 926 gammarids were included in the analyses (sample sizes per group on Fig. 1); they spent on average 38.0 ± 32.2% (mean ± standard deviation) of the test time under their refuge. Infected gammarids had on average 1.3 ± 0.8 cystacanths (ranging from 0 to 6) and 0.5 ± 0.7 acanthellae (ranging from 0 to 5). Among control gammarids (i.e., those that never received ES) of both experiments, gammarids infected with cystacanth(s) and those with mixed infections used the refuge less than uninfected gammarids (Z =  − 3.06, adjusted *P* = 0.0067 and Z =  − 3.15, adjusted *P* = 0.0098, respectively), but those infected with only acanthella(e) did not (Z = 1.21, adjusted *P* = 0.27). Gammarids infected with acanthella(e) only used their refuge more than those infected with cystacanth(s) only (Z = 2.51, adjusted *P* = 0.0182) and those with mixed infections (Z = 2.76, adjusted *P* = 0.0117). Gammarids with mixed and cystacanth(s)-only infections did not differ in refuge use (Z = 0.71, adjusted *P* = 0.48).

In both experiments, uninfected acutely stressed (AS) gammarids used the refuge more than the uninfected controls, whether they also received the chronic ES (“chronic stress” (CS); “low intensity” (LI): Z = 2.88, *P* = 0.0040; “high intensity” (HI): Z = 2.61, *P* = 0.0091) or not (“chronic control” (CC); LI: Z = 2.73, *P* = 0.0063; HI: Z = 2.08, *P* = 0.0378; Fig. 1). The CS/AC gammarids in the HI experiment also hid more than controls (Z = 2.14, *P* = 0.0320). However, there was no significant difference in refuge use between CS/AS and either CS/AC or CC/AS uninfected gammarids (Table S2).

**Figure 2:**
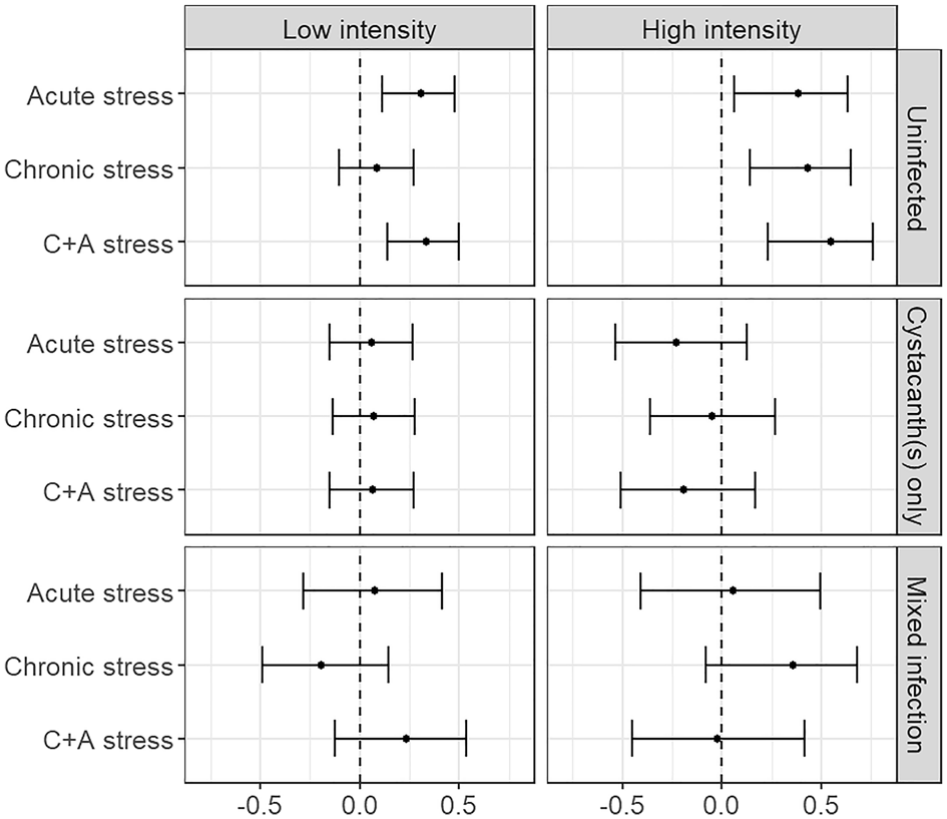
Effect sizes of refuge use of *Gammarus fossarum* for each electric-shock treatment compared to controls in both experiments, separated by infection statuses *with Pomphorhynchus tereticollis*. Points represent Cliff’s deltas and error bars represent confidence intervals. Positive values mean the focal group used the refuge more than controls of the same infection status, and negative values mean they used it less. *C* + *A:* “chronic and acute”.

**Figure 3:**
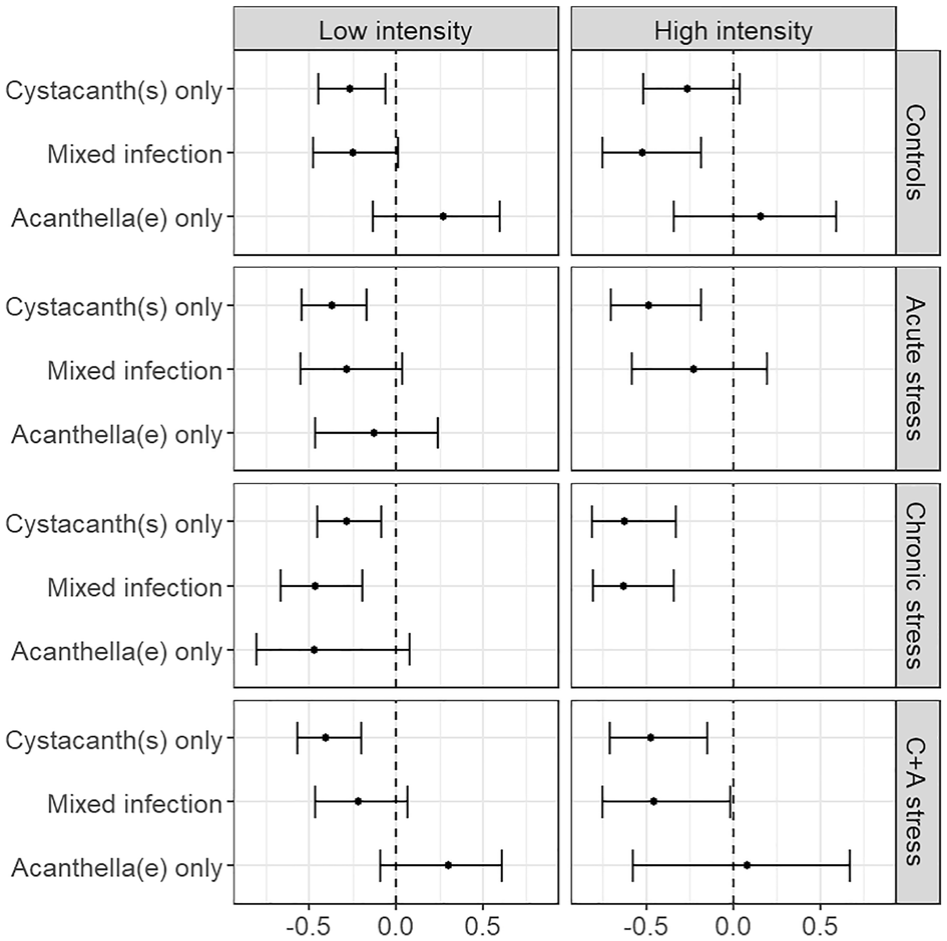
Effect sizes of refuge use of *Gammarus fossarum* for each infection status with *Pomphorhynchus tereticollis* compared to uninfected individuals in both experiments, separated by electric shock treatment. Points represent Cliff’s deltas and error bars represent confidence intervals. The effect sizes for the “acanthella(e) only” groups in the “acute stress” and “chronic stress” treatments in the “high intensity” experiment are not shown because the sample sizes were too low (N = 1). Positive values mean the focal group used the refuge more than uninfected individuals from the same treatment, and negative values mean they used it less. C + A: “chronic and acute”.

By contrast, refuge use by gammarids infected with cystacanth(s) did not differ among ES treatments (Fig. 1; test results in Table S2). The results were similar with gammarids harbouring mixed infections (cystacanths and acanthellae), except for the CS/AS gammarids of the LI experiment, which used the refuge more than CS/AC individuals (Z = 2.18, *P* = 0.0290). In all ES treatments of both experiments, gammarids with cystacanth(s) used the refuge less than their uninfected counterparts (CC/AS, LI: Z = 3.95, *P* < 0.0001; HI: Z = 4.00, *P* < 0.0001. CS/AC, LI: Z = 2.55, *P* = 0.0109; HI: Z = 3.47, *P* = 0.0005. CS/AS, LI: Z = 4.25, *P* < 0.0001; HI: Z = 4.08, *P* < 0.0001). The same was true of gammarids with mixed infections, except for the CS/AS individuals in the LI experiment (Table S2). In addition, CS/AS gammarids infected with only acanthella(e) used their refuge more than their CS/AC counterparts (Z = 2.73, *P* = 0.0063) and the CS/AS individuals infected with only cystacanth(s) (Z = 2.91, *P* = 0.0056). Finally, gammarids who received only one 10 × 15 V ES session the day before the test did not hide more than controls (N = 40 in each group, W = 878, *P*-value = 0.4523). Effect sizes are shown in Figs. 2 and 3. Potential confounding factors did not influence the results (Sup. Mat.).

## Discussion

Our experiment showed no anxiety-like response to ES in gammarids infected with *P. tereticollis* cystacanth(s), while confirming the triggering of such response in uninfected gammarids. In absence of ES exposure, basal anxiety levels were higher in uninfected controls than in cystacanth(s)-infected controls as previously reported in *G. fossarum*^29^, using refuge use as a proxy for anxiety-like state. We consider gammarids’ increased refuge use as a behavioural component of anxiety, as it is observed in the absence of familiar threatening cues (e.g. predator smell, moving shadow).

Among the uninfected individuals that received only the chronic treatment, only the higher-voltage treatment induced higher refuge use. This suggests that anxiety-like response is proportional to stimulus intensity, which was already evidenced in *G. fossarum*^29^ and in crayfish^37^. Contrasting responses of uninfected gammarids to “acute only” and “chronic only” treatments with the combined treatment did not provide evidence for habituation nor sensitization. Since gammarids which received only one “high intensity” ES session 24 h before the refuge use test did not show enhanced sheltering behaviour, we assume that the higher refuge use in gammarids of the “chronic, high intensity” treatment truly reflects a motivational change caused by chronic stress rather than a remanent effect of the last ES session.

We interpret the absence of anxiety-like response to ES in gammarids infected with *P. tereticollis* cystacanth(s) as a lack of avoidance/escape responsiveness to noxious stimulus, and therefore of specificity in the reversal of protective behavioural response by cystacanth. This may challenge the adaptive view of parasite manipulation at first sight. However, specificity in predation enhancement may not be necessary for manipulation to evolve in trophically transmitted parasites^9,10^. In addition, in the wild, predators may represent most of the threats faced by gammarids, which could make manipulation of general fear/anxiety circuitry more often beneficial (predation) than detrimental (predator-unrelated damages or death). It might also require more sophisticated mechanisms to manipulate specifically defence behaviour against suitable final host predators rather than general fear/anxiety-related pathways, thus, acquiring genes that allow to interfere with generalist and phylogenetically widespread pathways (such as serotonergic or glutamatergic pathways) might require less evolutionary steps and possibly be more likely. Finally, manipulation by acanthocephalans is multidimensional^38^, with some manipulated traits, such as geotaxis and phototaxis, showing some degree of specificity. For example, the bird acanthocephalans *Polymorphus minutus* inverts gammarids geotaxis but not phototaxis, while the fish acanthocephalans *P. laevis* and *P. tereticollis* invert phototaxis and not geotaxis or to a lesser extend^26,39^, although these parasites also increase predation by non-host invertebrates^40^. Therefore, while some manipulated traits, such as potentially impaired fear/anxiety circuitry, may seem to offer weak increase in parasite net fitness, their association with other manipulated traits may strengthen their fitness advantage.

Because their interests are partly divergent, parasites of different developmental stages might produce different effects on intermediate host anxiety-like behaviours^11^. In our experiments, gammarids infected with acanthella(e) behaved like uninfected ones in response to the combined acute and chronic ES treatment but their response to acute-only or chronic-only treatment was undifferentiable from uninfected or cystacanth-infected gammarids. This raises the questions of the mechanisms underlying parasite-induced modulation of anxiety-like responses at the neural level and their onset during parasite development. The fact that acanthella(e)-infected control gammarids used the refuge more than cystacanth(s)- and mixed-infected ones (although not more than uninfected controls) is congruent with a study on *P. laevis* that evidenced increased refuge use (and lower predation) in acanthella(e)-infected *G. pulex*, contrary to cystacanth(s)-infected individuals^11^. In line with the ‘switcher paradigm’^12^, increased antipredator defence by pre-infective developmental stage could be as important in host manipulation as decreased antipredator defence by the parasite stage infective to final hosts. Indeed, parasite fitness is expected to increase with parasite-induced predation enhancement at the latter stage, and with predation suppression at the former stage^41^. Maintaining host’s ability to respond to a noxious stimulus with a defensive/protective behaviour would therefore be adaptive for the parasite. Investigation should be conducted further to confirm the observed trends, as our study was not designed to evaluate specifically the effect of acanthellae on host behaviour and lacks power because of the low sample sizes of acanthella(e)-only infected groups. Finally, the behaviour of individuals co-infected with both stages was comparable to that of cystacanth(s)-infected hosts. We did not find differences between the mixed-infection categories. However, our results do not allow us to draw definitive conclusions on the outcome of potential conflicts between different co-infecting life stages, because the numbers and relative proportions of parasites of each stage were very variable and sample sizes in each category were low. In addition, acanthellae were of different ages and probably varied in their manipulation potential, assuming that manipulation factor(s), as defined by^14^, have had more time to accumulate and to induce effects in individuals infected by older acanthellae.

The lack of response to ES of gammarids infected with *P. tereticollis* cystacanth(s) could be due to impaired nociception. However, all infected gammarids responded to ES by tail-flicking during the two seconds that each pulse lasted, which suggests that peripheral perception of the noxious stimulus was preserved. The lack of behavioural response compatible with anxiety-like state under acute/chronic stress in infected gammarids may then result from parasite intervention at any of these steps: inappropriate processing of the nociceptive signal, altered decision making process, or impaired translation into behavioural response. Immunohistological and biochemical studies investigating neural processes are now needed to obtain a more precise picture of altered mechanisms.

Compared to research on mammals, few studies have sought to study emotions—defined as “internal central nervous system states that give rise to physiological, behavioural and cognitive responses”^42,43^—in invertebrates (reviewed in^19^). Yet, the alteration of emotional state could impact multiple traits, potentially explaining multidimensionality in PIPA and even a form of infection syndrome^1^. The generalization of the fear/anxiety-reducing effect to stressors that are not directly useful for parasite transmission (i.e., cues unrelated to predators) also gives further credit to the hypothesis of parasites affecting general neuromodulator pathways^15,16^. As in humans and other vertebrates^44,45^, serotonin seems to be an important modulator of fear and anxiety in invertebrates^32,37,46^ and would make an ideal candidate pathway for manipulation^15^. Further studies aimed at identifying manipulation factors are needed to determine whether and how parasites interfere with serotonergic system and other emotion-related neuromodulatory pathways, such as GABAergic^47^ and glutamatergic systems^36^. More importantly, such studies should take a multisystemic view of brain neurotransmitter homeostasis, to address whether unbalanced levels of glutamate/GABA and upstream modulation by serotonin/dopamine are responsible for anxiety-like behaviours^29^. Acanthocephalan infections in gammarids impact several behavioural traits with varying effect sizes^25,28^, making these crustacean species and their parasites a particularly valuable model system for neuro-ecological studies of anxiety in invertebrate and of parasite-induced modulation of related cognitive abilities.

In conclusion, we showed that the effect of an acanthocephalan parasite on its amphipod host behaviour is not restricted to antipredator defence, but also encompasses anxiety-like reaction to a noxious stimulus. In the future, impaired associative learning should be addressed, in particular aversive learning. In fact, antipredator behaviour might be partially (olfactorily) learnt in some aquatic invertebrates^48,49^, such that the alteration of threat-sensitive learning of predator avoidance might also be a powerful way for trophically transmitted parasites to achieve their transmission to final hosts. Our results further encourage focussing on biogenic amines modulating emotions, in particular anxiety, in mechanistic studies aimed at identifying manipulative parasites’ specific targets and manipulation factors. The exploration of other aspects of infected host cognitive abilities such as associative learning might as well be a promising route.

## Methods

### Organisms

Once a month from October 2021 to February 2022, we collected *P. tereticollis*-infected and uninfected *G. fossarum* individuals in the river Chiron in Burgundy, France (47° 27′ 25.7"N, 5° 18′ 21.2"E). They were maintained in large tanks filled with a mix of oxygenated dechlorinated ultraviolet-treated water (conditioned water; CW) and water from their river, in a room at 16 °C with a 12L:12D photoperiod. Rocks were available as substrate and refuge, and gammarids were fed with dry elm leaves and chironomid larvae. Water was partly renewed every week. Gammarids were acclimatized to the room conditions for at least three weeks before taking part in the experiment.

### Experimental procedure

#### Treatments: acute and chronic stress using a semi-automated electric shock device

Gammarids were placed in plastic containers (150 × 105 mm, height: 60 mm) with 400 ml of CW by batches of 15 to 20 individuals, separated by treatments and according to the presence or absence of cystacanths. The cystacanths and old acanthellae of *P. tereticollis* form orange spots visible through *G. fossarum* exoskeleton, which allows straightforward diagnosis in living hosts; younger acanthellae have a lighter colouration and their presence can be assessed after host dissection. They were provided a small stone and half a terracotta saucer as refuges, and an elm leaf as food. Exposure to ES was done using a semi-automatic ES device (ESD) made of a rectangular piece of 3D-printed polymer containing two electrodes connected to an electronic driver^30^, that fitted inside the above-mentionned plastic containers.

In the “low intensity” (LI) stress treatment, once a day for 6 days at varying hours, the “chronically stressed” (CS) gammarid groups were placed in the ESD that delivered an ES program consisting in ten 9 V pulses of 2 s administered every minute for 10 min. Water was daily renewed at the time of the ES treatment. “Chronic controls” (CC) were not placed in ESDs but their water was renewed at the same time. Chronic treatment ended the day before behavioural tests (see below). On the seventh day, the “acutely stressed” (AS) gammarids were placed individually in plastic containers holding 200 ml of CW, inside an ESD. They were given 5 min to acclimatize before the ES program started. “Acute controls” (AC) were in the same conditions, except that they were not placed in ESDs, and their refuge was moved to the centre of their container. When the ES program ended, we removed the ESDs and placed the refuges back to the top edge of each container.

In the “high intensity” (HI) stress treatment, the same protocol was applied to another set of gammarids except that a higher number of pulses and/or voltage was used. The HI CS treatment consisted in twenty 9 V pulses of 2 s administered every 30 s for 10 min once a day for the three first days, and then ten 15 V pulses of 2 s every minute for 10 min once a day for the last three days. On the seventh day, the AS gammarids received the 10 × 15 V ES program before the refuge use test.

Finally, to differentiate between a truly chronic effect of the CS treatment and a persistent effect of the last ES session, we exposed uninfected gammarids to a 10 × 15 V ES session and conducted refuge use tests 24 hs later; controls were kept in the same conditions, but they did not receive ES. Although we configured the ESD to administer 9 V or 15 V pulses, effective voltage inside the device was lower and peaked at around 4 V and 9 V, respectively (measured with Voltcraft Digital Multimeter VC830).

#### Refuge use test

Refuge use tests were conducted on a bench receiving light between 90 and 130 lx. The choice of dimmed light (around sunset/sunrise values) was motivated by the decreased photophobia of uninfected gammarids at low illuminance^27^, thus allowing the detection of a possible increase in photophobia in stressed gammarids. Five minutes after the end of the AS ES program (acclimation time), we started recording gammarids’ position every thirty seconds for ten minutes (focal and time sampling). Each gammarid’s score, thereafter abbreviated “RUs” was calculated as the sum of observations during which the gammarid was inside the refuge (from 0 to 20). Refuge use tests were generally conducted in blocks of 12 gammarids in individual plastic containers, each of the 8 treatments being represented by at least one individual in each block (with a few exceptions). After each refuge use test, gammarids were sedated in a 0.6 g/l MS222 solution until they stopped moving^30^, weighted and dissected to count the number of cystacanth(s) and acanthella(e).

### Statistical analyses

All statistical analyses were performed using R version 4.2.1 on RStudio 2022.07.1^50^. We ran Kruskal–Wallis tests using an explanatory variable built as the interaction between infection status and ES treatment to evaluate their combined effect on gammarid RUs. We first separated infection status in six categories: “uninfected”, “cystacanth(s) only”, “acanthella(e) only”, “mixed infection with the same number of cystacanth(s) and acanthella(e)”, “mixed infection with more acanthella(e)” and “mixed infection with more cystacanth(s)”. To correct for multiple testing when identifying significant comparisons, we applied the Benjamini–Hochberg procedure using only relevant comparisons (comparing different treatments within an infection status or different infection statuses within a treatment) and a false discovery rate of 0.05. We conducted these tests on the LI and HI experiments separately. As the three “mixed infection” statuses had low sample sizes and did not significantly differ in RUs (Table S2), we pooled them into a single “mixed infection” category and reconducted the tests. As sample sizes of the “acanthella(e) only” group was low in the HI experiment, we removed it from the test and compared only the three remaining infection status categories. Cliff’s deltas were computed as a measure of effect size for each treatment (CC/AC, thereafter called “controls”, versus CS/AC, CC/AS and CS/AS) separately for each parasitic status and ES program, and for each infection status (uninfected versus cystacanth(s)-infected, acanthella(e)-infected and mixed-infected), separately for each treatment and ES program.

We also ran a Dunn’s test with Benjamini–Hochberg correction on a third sub-dataset containing only the controls of both experiments, to compare basal refuge use between the four infection categories. We compared RUs of gammarids that received the 10 × 15 V treatment only once 24 hs before refuge use tests and their associated controls to test for a remanence effect in the HI chronic treatment, using a Wilcoxon test. Verifications for confounding factors are reported in Sup. Mat.

### Ethics

The study complies with the rules of ethics as prescribed by the French legislation and the University of Bourgogne Franche-Comté.

## Supplementary Information

Supplementary Information 1.

Supplementary Table S1.

Supplementary Table S2.

## Data Availability

Raw data are available at https://doi.org/10.6084/m9.figshare.21571662.v1.
