# Supplementary material for: Infection with an acanthocephalan helminth reduces anxiety-like behaviour in crustacean host
Source: Sci Rep. 2022 Dec 15;12:21649. doi: 10.1038/s41598-022-25484-9 (PMC9755125; doi:10.1038/s41598-022-25484-9)
Supplement: Supplementary file 1 — Supplementary Information 1. [file 41598_2022_25484_MOESM1_ESM.docx]

# Infection with an acanthocephalan helminth reduces anxiety-like behaviour in crustacean host

# Supplementary material

Camille-Sophie Cozzarolo* (0000-0002-9056-8622), Marie-Jeanne Perrot-Minnot* (0000-0003-3412-4282)

* Biogéosciences, UMR 6282 CNRS, université Bourgogne Franche-Comté, 6 boulevard Gabriel, 21000 Dijon, France

Correspondence: [camille-sophie.cozzarolo@u-bourgogne.fr](mailto:camille-sophie.cozzarolo@u-bourgogne.fr)

# Statistical analysis: potential confounding factors

Infected gammarids were generally smaller than uninfected gammarids; to evaluate which part of the variance in refuge use could be attributed to gammarid weight rather than their infection status, we built a generalized linear model with a quasibinomial distribution using R package lme4 (Bates et al. 2014). The proportion of time spent under the refuge (RUp) was calculated as the refuge use scores (RUs) divided by 20. We fitted RUp as response variable and gammarid’s weight, as well as the interaction between infection status and acute treatment, and between the infection status, the chronic treatment and the experiment as explanatory variables. The model structure was based on results from treatment comparisons described at in the *statistical analyses* section of the main text. We fitted this model on a sub-dataset excluding individuals infected only by acanthella(e) (low sample size) to avoid convergence issues. The significance of a variable was assessed by comparing residual deviances of the models before and after dropping the focal variable, using the F-test. As there is no method to fit generalized linear mixed models with quasibinomial error distribution, we separately checked for other potential confounding effects. The effect of gammarids’ capture date as well as of the date and time of tests as categorical variables on RUs were assessed with Kruskal-Wallis tests, and Dunn’s tests were also used to identify significant comparisons. The effects of position on the bench during tests, test day (from 1 to 107, corresponding number of days after the first test day) and time as an ordinal variable (from 1 to 5, corresponding to 2-hour time slots, from 7am-9am to 3pm-5pm) on refuge use scores were evaluated with Spearman’s correlations.

# Results

Weight was positively correlated with refuge use (Odds ratio: 1.017, 95% CI: 1.004-1.030; F_1_ = 5.50, P = 0.0192), but the interaction between infection status and acute treatment (F_3_ = 11.85, P < 0.0001) as well as the interaction between the infection status, the chronic treatment and the intensity (F_9_ = 2.78, P = 0.0032) stayed significant. Neither gammarids’ capture date (χ_5_^2^ = 3.196, P = 0.670), categorical time (χ_5_^2^ = 52.741, P = 0.407), position on the bench (ρ_S_ = 0.047, P = 0.642), test day (ρ_S_ = -0.005, P = 0.959) or ordered time slots (ρ_S_ = -0.101, P = 0.315) were associated with refuge use scores of uninfected controls. Date was significantly associated with refuge use scores (χ_16_^2^ = 26.679, P = 0.045): especially high refuge use scores were observed in uninfected controls on December 8^th^, 2021, that were significantly higher than three other dates.

# Table captions

**Table S1. Results from Dunn test on *Gammarus fossarum* refuge use with Benjamini-Hochberg multiple comparisons adjustment in the “low intensity” experiment.** Only relevant comparisons of refuge use scores between groups (electric shock treatments and *Pomphorhynchus tereticollis* infection status) are shown. A: only acanthella(e); C: only cystacanth(s); A=C: same number of acanthella(e) and cystacanth(s); A>C: more acanthella(e) than cystacanth(s); C>A: more cystacanth(s) than acanthella(e); U: uninfected.

**Table S2. Results from Dunn test on refuge use scores of *Gammarus fossarum* with Benjamini-Hochberg multiple comparisons adjustment.** Only relevant comparisons of refuge use scores between groups (intensity, electric shock (ES) treatments and *Pomphorhynchus tereticollis* infection status) are shown. LI: “low intensity” treatment; HI: “high intensity” treatment.

# References

Bates D, Mächler M, Bolker B, Walker S. 2014 Fitting Linear Mixed-Effects Models using lme4. *Submitt. to J. Stat. Softw.* **67**. (doi:10.18637/jss.v067.i01)
